# CAR19/22 T‐Cell Cocktail Therapy Combined With Autologous Stem Cell Transplantation for a Patient With Mosaic *TP53* Mutation and 17p Deletion

**DOI:** 10.1111/jcmm.70715

**Published:** 2025-07-13

**Authors:** Kefeng Shen, Qiang Gao, Jia Gu, Caixia Chen, Wei Mu, Jiachen Wang, Xia Mao, Shugang Xing, Yajun Li, Min Xiao, Yi Xiao

**Affiliations:** ^1^ Department of Hematology, Tongji Hospital, Tongji Medical College Huazhong University of Science and Technology Wuhan Hubei China; ^2^ Immunotherapy Research Center for Hematologic Diseases of Hubei Province Wuhan Hubei China; ^3^ Department of Lymphoma and Hematology The Affiliated Cancer Hospital of Xiangya School of Medicine, Central South University/Hunan Cancer Hospital Changsha Hunan China; ^4^ Key Laboratory of Vascular Aging, Ministry of Education Huazhong University of Science and Technology Wuhan Hubei China

**Keywords:** Burkitt's lymphoma, CAR‐T therapy, durable remission, mosaic *TP53* mutation, multiple‐hit *TP53* aberrations

## Abstract

Although most cases of Burkitt lymphoma (BL) respond well to chemotherapy, relapsed or refractory BL, particularly in the presence of *TP53* aberrations, presents a significant therapeutic challenge. *TP53* germline or mosaic mutations, though rare, are clinically relevant in lymphomas as they persist in both lymphoma cells and autologous chimeric antigen receptor (CAR)‐T cells, potentially influencing treatment outcomes. Here, we report the first documented case of a *TP53* mosaic mutation and a 17p deletion in an adult patient with refractory BL. After four cycles of unsuccessful standard induction chemotherapy, the patient received CAR‐T cell therapy combined with autologous stem cell transplantation. The comprehensive treatment plan included radiotherapy bridging, sequential infusions of CD19 and CD22 CAR‐T cells, and maintenance therapy with chidamide, obinutuzumab, sintilimab, and azacitidine. Two months postinfusion, the patient achieved complete remission, which has been sustained for 24 months to date. This case indicates that, despite the challenges posed by multiple‐hit *TP53* aberrations in refractory BL, a multifaceted therapeutic approach can achieve positive outcomes. Furthermore, based on previous studies and our findings, we speculate that *TP53* alterations may play a biphasic role in this patient's CAR‐T therapy: in lymphoma cells, *TP53* alterations impair CAR‐T cell cytotoxicity, while in CAR‐T cells, they may promote enhanced expansion and persistence. This potential dual role might help explain why *TP53* deficiency does not universally affect the efficacy of CAR‐T therapy in certain studies.

## 1 Introduction

Although most Burkitt's lymphoma (BL) cases are extremely sensitive to intensive chemotherapy and can potentially be cured, the treatment of relapsed or refractory BL remains a significant challenge [1]. Several factors may increase the risk of relapse or refractoriness, including advanced disease stage, high lactate dehydrogenase (LDH) levels, and adverse molecular abnormalities [2]. Patients with *TP53* aberrations, including *TP53* mutations and/or 17p deletions, generally fall into a high‐risk group and have limited treatment options. Encouragingly, chimeric antigen receptor (CAR)‐T cell therapy may offer a glimmer of hope as it emerges as a promising and innovative treatment for high‐risk hematologic malignancies [3, 4, 5]. Additionally, our previous study demonstrated the efficacy and safety of CAR19/22 T‐cell cocktail therapy in patients with aggressive B‐cell lymphoma who harbour *TP53* alterations [6].

*TP53* mosaic mutation is relatively rare and has distinct features compared to *TP53* germline and somatic mutations [7]. To our knowledge, *TP53* mosaic mutation has not been previously reported in lymphomas. Here, we report the first documented case of a *TP53* mosaic mutation and a 17p deletion in a patient with lymphoma. We aim to present a successful treatment experience and provide new insights into managing similar patients.

## 2 Case Presentation

A 41‐year‐old man was diagnosed with high‐risk BL, Stage IV (Ann Arbor system), in June 2022. The patient presented with a significantly elevated LDH level of 1563 U/L at diagnosis, which was six times the upper normal limit, but with no evidence of bulky disease (> 10 cm), as well as central nervous system or bone marrow involvement. Fluorescence in situ hybridisation confirmed the presence of *MYC* translocation, 17p‐ (*TP53* deletion), and 9p‐ (*CDKN2A* deletion). Next‐generation sequencing (NGS) revealed a mutational profile consistent with BL, including *TP53* p.R273H (86.7%), *DDX3X* p.D97Gfs*2 (94.4%), *MYC* p.E54D (55.7%), and *FOXO1* p.R19W (51.9%). Of note, the variant allele frequency (VAF) of the *TP53* mutation exceeded 50%, which was primarily attributed to the allelic *TP53* deletion.

At diagnosis, the patient had a high tumour burden and poor general condition. To reduce the tumour burden and mitigate the risk of tumour lysis syndrome, preconditioning chemotherapy with the CHP regimen was administered. Over the following 2 months, the patient was initiated on standard induction chemotherapy, comprising one cycle of the R‐CODOX regimen and one cycle of the R‐MAD regimen. However, the disease responded poorly, and the patient's condition progressively worsened. The patient then received two cycles of chemotherapy with the PD1‐GDCE and G‐MINE regimens but failed to achieve even partial remission. Comprehensive details of the patient's treatment history prior to CAR‐T therapy are summarised in Table S1. Given the patient's primary refractory high‐risk BL with poor prognostic factors, including multihit *TP53* alterations, the multidisciplinary lymphoma team recommended CAR19/22 T‐cell cocktail therapy combined with autologous stem cell transplantation (ASCT), based on results from our previous studies [4, 5, 6].

**FIGURE 1:**
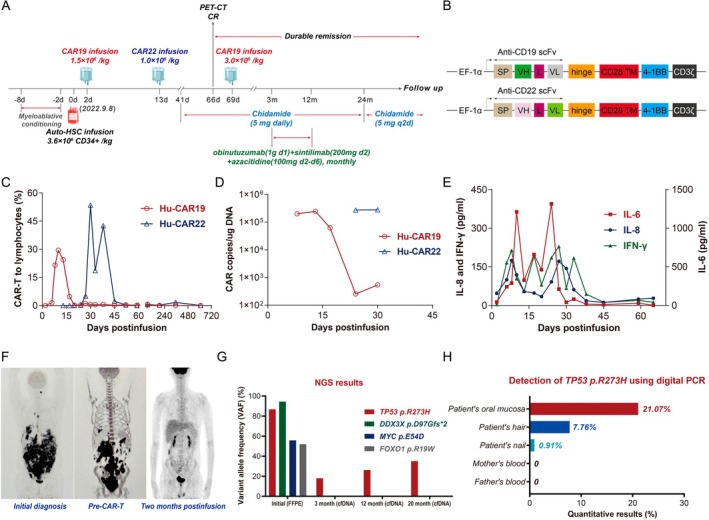
Clinical trajectory of the patient and associated laboratory and imaging tests. (A) Patient timeline illustrating the sequential infusion protocol of CAR19/22 T‐cells, combined with autologous stem cell transplantation (ASCT) and subsequent maintenance therapy. Following myeloablative conditioning with a personalised regimen (idarubicin 20 mg on Day −8; bendamustine 285 mg on Days −8 to −7; fludarabine 75 mg on Day −6; and 50 mg on Days −5 to −3; cytarabine 4 g on Days −6 to −5; and melphalan 266 mg on Day −2), a total of 3.6 × 10^6^ CD34^+^ cells/kg was infused on Day 0. Subsequently, 1.5 × 10^6^ CD19 CAR‐T cells/kg and 1.0 × 10^6^ CD22 CAR‐T cells/kg were administered on Days +2 and +13, respectively. To further consolidate the therapeutic effect, the patient received an additional infusion of 3.0 × 10^6^ CD19 CAR‐T cells/kg on Day +69. Following CAR‐T therapy, a multidrug combination strategy was adopted for the maintenance treatment, including chidamide, obinutuzumab, sintilimab, and azacitidine. (B) Schematic diagram of human anti‐CD19 and anti‐CD22 CAR constructs (provided by Hebei Senlangbio). (C–D) Monitoring of CAR‐T cell populations and human CAR transgene copy numbers in peripheral blood using flow cytometry and digital PCR, respectively, revealed relatively strong peak expansion and prolonged persistence of CAR‐T cells. The CAR19 and CAR22 exhibited a maximal CAR transgene expansion (*C*_*max*_) of 2.4 × 10^5^ and 2.8 × 10^5^ copies/μg genomic DNA, and a CAR‐T cell persistence (*T*_*last*_) of 6 and 20 months, respectively. (E) Temporal changes in IL‐8, IFN‐γ, and IL‐6 levels following infusion. (F) Whole body ^18^F‐FDG PET/CT imaging performed at initial diagnosis, pre‐CAR‐T disease assessment, and 2 months postinfusion. (G) Mutational profiling of the patient's lymphoma revealed by NGS on FFPE samples at initial diagnosis, with serial tracking of these mutations using ultra‐sensitive cfDNA NGS at 3, 12, and 20 months postinfusion. (H) The *TP53* p.R273H mutation was analysed in the parents' peripheral blood, as well as in the patient's oral mucosa, hair, and nails using digital PCR, confirming its mosaic origin. Auto‐HSC, autologous haematopoietic stem cell; CAR, chimeric antigen receptor; cfDNA, cell‐free DNA; CR, complete response; d, day; FFPE, formalin‐fixed paraffin‐embedded; IFN‐γ, interferon‐γ; IL, interleukin; L, linker; m, month; NGS, next‐generation sequencing; PET‐CT, positron emission tomography‐computed tomography; scFv, single chain variable fragment; SP, signal peptide; TM, transmembrane domain; VH, variable H chain; VL, variable L chain.

The sequential infusion protocol of CAR19/22 T‐cells, combined with ASCT and subsequent maintenance therapy, is illustrated in Figure 1A. Details on the structure (Figure 1B), manufacturing, and preparation of the third‐generation CAR‐T cells simultaneously expressing CD28 and 4‐1BB costimulatory domains have been described previously [3, 4]. Monitoring of CAR‐T cell kinetics revealed relatively strong peak expansion and prolonged persistence (Figure 1C,D, and Table S2). Serum IL‐6 levels (> 1000 pg/mL) peaked on Days +10 and +24 (Figure 1E). The patient experienced two episodes of cytokine release syndrome (CRS), both of which were alleviated by treatment with tocilizumab and supportive care. Throughout the treatment course, no immune effector cell‐associated neurotoxicity syndrome (ICANS) was observed.

**FIGURE 2:**
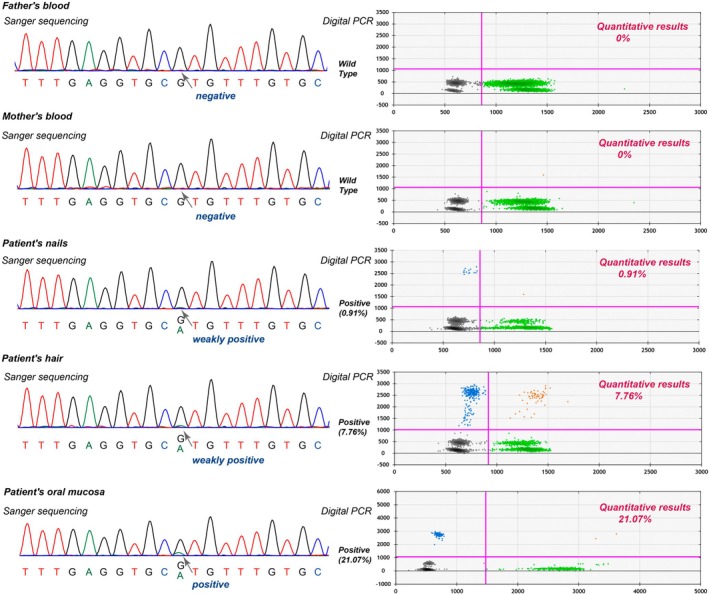
The *TP53* c.818G>A (p.R273H) mutation was detected using Sanger sequencing and digital PCR to confirm its mosaic origin. The left panel displays Sanger sequencing chromatograms, showing negative results for the parents' peripheral blood and positive or weakly positive results for the patient's oral mucosa, hair, and nails (dark grey arrow). The right panel presents digital PCR scatter plots, illustrating the quantitative results of the *TP53* mutation in the aforementioned samples. The magenta lines represent the fluorescence thresholds used to distinguish positive from negative droplets. Grey droplets are negative, while orange‐red droplets are positive for both *POP4* and the *TP53* mutation. Green droplets are positive only for *POP4* (reference gene, VIC fluorophore), and blue droplets are positive only for the *TP53* mutation (FAM fluorophore).

Two months after infusion, the patient achieved complete metabolic remission (Figure 1F). At 3 months, an ultra‐sensitive NGS (VAF sensitivity, 0.3%) was performed on cell‐free DNA (cfDNA) from the patient's blood sample to track minimal residual disease (MRD). Notably, the lymphoma‐associated mutations in *DDX3X*, *MYC*, and *FOXO1* identified at diagnosis became undetectable, while the *TP53* p.R273H mutation remained positive, with the VAF decreasing from 86.7% to 17.9% (Figure 1G). We integrated various results and ruled out the possibility of tumour residue. Furthermore, we detected the *TP53* p.R273H mutation in genomic DNA from the parents' blood and the patient's oral mucosa, hair, and nails using Sanger sequencing and digital PCR, concluding that the patient harboured a mosaic *TP53* p.R273H mutation (Figures 1H and 2).

As of September 2024, the patient has been under regular follow‐up for 24 months. PET‐CT and cfDNA NGS were performed to assess treatment response. The patient remains in durable complete remission and achieves sustained NGS MRD negativity with a sensitivity of 0.3% (Figure 1G). Invariably, the *TP53* p.R273H mutation remains detectable due to its mosaic origin.

## 3 Discussion

Somatic, germline, and mosaic mutations are all types of *TP53* mutations that can occur in the body. Mosaic mutations occur during early embryonic development, leading to the body carrying two or more genetically distinct cell lineages [8]. Mosaic *TP53* mutations, though rare, are clinically relevant in lymphomas as they persist in both lymphoma cells and autologous CAR‐T cells, potentially influencing treatment outcomes.

*TP53* aberrations have historically been reported to be predictive of dismal prognosis in patients with aggressive lymphoma. While CAR‐T therapy offers potential benefits for these patients, its failure due to tumour‐extrinsic and/or intrinsic factors remains a matter of concern. The clinically significant question of whether *TP53* deficiency in lymphoma cells and/or autologous CAR‐T cells impacts CAR‐T therapy, and if so, how, remains largely unaddressed. A recent study by Shouval et al. described *TP53* aberrations in lymphoma cells as a tumour‐intrinsic factor contributing to CAR‐T therapy failure [9]. Utilising gene expression profiling, they indirectly demonstrated that *TP53* alterations impair CAR‐T cell cytotoxicity by disrupting various cellular processes.

However, the clinical question of whether *TP53* alterations in CAR‐T cells enhance their expansion, persistence, or antitumor efficacy remains largely unexplored to date. Prior studies have shown that loss‐of‐function mutations in *TET2* and *DNMT3A* in CAR‐T cells lead to enhanced proliferative capacity and potent antitumor activity [10]. *TP53* alterations are well known to confer survival advantages to cells, such as increased proliferation, evasion of apoptosis, and drug resistance. Thus, CAR‐T cells harbouring *TP53* alterations may exhibit comparable effects to those driven by *TET2* and *DNMT3A* mutations. This hypothesis is partially supported by recent research from Roselle et al., which demonstrated that modulating the p53 signalling network with Δ133p53α enhances CAR‐T cell function [11]. In a prior study, we reported the case of an adult lymphoma patient who carried a germline *TP53* p.R273H mutation and received two rounds of autologous CAR‐T cell therapy [12]. After each infusion, the *TP53*‐deficient CAR‐T cells exhibited rapid exponential expansion and sustained persistence. Here, we present a novel case of a new patient harbouring a mosaic *TP53* p.R273H mutation, and the CAR‐T cells with *TP53* disruption demonstrated similar results. Together, these findings suggest that intrinsic *TP53* alterations in CAR‐T cells may enhance their expansion and persistence. This potential dual role might help explain why *TP53* deficiency does not universally affect the efficacy of CAR‐T therapy in certain studies. However, further functional studies are needed to confirm this hypothesis.

This case also suggests that a multifaceted therapeutic approach can achieve positive outcomes despite the challenges posed by multi‐hit *TP53* aberrations in lymphoma. First, radiotherapy was used as a bridging regimen to control disease progression before CAR‐T infusion. Second, sequential infusion of CAR19/22 T‐cells combined with ASCT helped reduce relapse and improve outcome. Third, histone deacetylase inhibitors, PD‐1 blockade, and hypomethylating agents may enhance CAR‐T cell function and improve prognosis to some extent as maintenance therapy following CAR‐T administration [13, 14]. Lastly, the risk of secondary primary malignancies after CAR‐T therapy is approximately 4.3% [15]. Moreover, this patient has Li‐Fraumeni syndrome resulting from a mosaic *TP53* mutation. The combination of these two factors significantly increases the patient's risk of developing a second tumour. Therefore, vigilant measures for cancer prevention and surveillance are essential.

## Author Contributions

**Kefeng Shen:** conceptualization (lead), data curation (equal), formal analysis (equal), visualization (lead), writing – original draft (lead), writing – review and editing (lead). **Qiang Gao:** data curation (equal), methodology (equal), validation (equal). **Jia Gu:** data curation (equal), formal analysis (equal), validation (equal). **Caixia Chen:** data curation (equal), formal analysis (equal), validation (equal). **Wei Mu:** methodology (equal), validation (equal). **Jiachen Wang:** data curation (equal), investigation (equal), resources (equal). **Xia Mao:** data curation (equal), formal analysis (equal), validation (equal). **Shugang Xing:** data curation (equal), investigation (equal), software (equal). **Yajun Li:** data curation (equal), methodology (equal), resources (equal). **Min Xiao:** conceptualization (lead), funding acquisition (equal), project administration (lead), supervision (lead). **Yi Xiao:** conceptualization (lead), funding acquisition (lead), project administration (equal), resources (equal), supervision (lead).

## Consent

Informed consent was obtained from both the patient and their family members.

## Conflicts of Interest

The authors declare no conflicts of interest.

## Supporting information


**Table S1.** Patient’s chemotherapy, targeted therapy, and radiotherapy records before CAR‐T infusion.
**Table S2.** Monitoring of CAR‐T cell kinetics and serum cytokine levels in peripheral blood postinfusion.

## Data Availability

The data that support the findings of this study are available on request from the corresponding author.
