# Supplementary material for: CAR19/22 T‐Cell Cocktail Therapy Combined With Autologous Stem Cell Transplantation for a Patient With Mosaic TP53 Mutation and 17p Deletion
Source: J Cell Mol Med. 2025 Jul 13;29(13):e70715. doi: 10.1111/jcmm.70715 (PMC12256272; doi:10.1111/jcmm.70715)
Supplement: Supplementary file 1 — Table S1. Patient’s chemotherapy, targeted therapy, and radiotherapy records before CAR‐T infusion. Table S2. Monitoring of CAR‐T cell kinetics and serum cytokine levels in peripheral blood postinfusion. [file JCMM-29-e70715-s001.docx]

**Supplementary material**

Table S1. Patient's chemotherapy, targeted therapy, and radiotherapy records before CAR-T infusion.

|  | **Start time** | **Treatment Regimen** | **Response assessment** |
| --- | --- | --- | --- |
| 1 | 2022.06.02 | ***CHP pre-chemotherapy (to reduce tumor burden)***: Cyclophosphamide 1.2g, Doxorubicin 40mg, Dexamethasone 80mg |  |
| 2 | 2022.06.17 | ***R-CODOX chemotherapy***: Rituximab 700mg, Cyclophosphamide 1.9g, Doxorubicin 40mg, Vincristine 2mg |  |
| 3 | 2022.07.05 | ***R-MAD chemotherapy***: Rituximab 700mg, Methotrexate 2g, Cytarabine 1.9g, Dexamethasone 20mg | ***PD (Progressive Disease)*** |
| 4 | 2022.07.14 | ***Rituximab*** 700mg |  |
| 5 | 2022.07.22 | ***PD1-GDCE chemotherapy***: Tislelizumab 200mg, Gemcitabine 1.5g, Dexamethasone 20mg, Cyclophosphamide 0.4g, Etoposide 190mg |  |
| 6 | 2022.07.27 | ***Inotuzumab ozogamicin*** 1mg |  |
| 7 | 2022.08.05 | ***G-MINE chemotherapy***: Ortolizumab 1g, Mitoxantrone 35mg, Ifosfamide 2g, Etoposide 0.1g | ***PD (Progressive Disease)*** |
| 8 | 2022.08.21 | ***Inotuzumab ozogamicin*** 1mg |  |
| 9 | 2022.08.23 | ***Abdominal radiotherapy***, totaling 9 Gy in 5 fractions |  |

Table S2. Monitoring of CAR-T cell kinetics and serum cytokine levels in peripheral blood post-infusion.

| *Date* | *Days post-infusion* | *CAR19 T cells to lymphocytes (%) using flow cytometry* | *CAR19 transgene copy numbers using digital PCR (copies/µg genomic DNA)* | *CAR22 T cells to lymphocytes (%) using flow cytometry* | *CAR22 transgene copy numbers using digital PCR (copies/µg genomic DNA)* | *IL-6 (pg/ml)* | *IL-8 (pg/ml)* | *IFN-γ (pg/ml)* |
| --- | --- | --- | --- | --- | --- | --- | --- | --- |
| 2022/09/06 | 0 |  |  |  |  |  |  |  |
| 2022/09/08 | 2 | 0.00% | / | / | / | 43.31 | 46.90 | 3.77 |
| 2022/09/12 | 6 | 1.49% | / | / | / | 241.34 | 99.89 | 166.40 |
| 2022/09/14 | 8 | 21.53% | 2.00E+05 | / | / | 289.81 | 174.57 | 214.03 |
| 2022/09/16 | 10 | 29.46% | / | / | / | 1212.44 | 118.71 | 104.22 |
| 2022/09/19 | 13 | 24.41% | 2.44E+05 | 0.00% | / | 326.79 | 54.16 | 54.63 |
| 2022/09/23 | 17 | 4.80% | 6.23E+04 | 0.00% | / | 659.85 | 48.27 | 192.08 |
| 2022/09/26 | 20 | 0.19% | / | 0.00% | / | 462.35 | 34.44 | 79.94 |
| 2022/09/30 | 24 | 0.39% | 2.54E+02 | 0.16% | 2.75E+05 | 1316.00 | 91.70 | 185.37 |
| 2022/10/03 | 27 | 1.09% | / | 5.06% | / | 211.33 | 171.67 | 229.09 |
| 2022/10/06 | 30 | 0.59% | 5.39E+02 | 53.52% | 2.78E+05 | 41.06 | 143.46 | 66.93 |
| 2022/10/09 | 33 | 0.49% | / | 18.62% | / | 81.74 | 61.33 | 184.18 |
| 2022/10/14 | 38 | 0.57% | / | 42.56% | / | 17.23 | 18.28 | 44.08 |
| 2022/10/21 | 45 | 0.19% | / | 2.27% | / | 10.69 | 11.56 | 12.00 |
| 2022/10/28 | 52 | 0.38% | / | 0.29% | / | / | / | / |
| 2022/11/04 | 59 | 0.18% | / | 0.00% | / | 29.42 | 24.45 | 19.33 |
| 2022/11/10 | 65 | 0.68% | / | 0.48% | / | 2.04 | 28.06 | 12.47 |
| 2023/03/13 | 188 | 0.07% | / | 0.23% | / | / | / | / |
| 2023/08/26 | 354 | 0.00% | / | 1.67% | / | / | / | / |
| 2024/05/08 | 610 | 0.00% | / | 0.04% | / | / | / | / |

Day 0, autologous hematopoietic stem cell infusion; Day 2, CAR19 infusion; Day 13, CAR22 infusion; /, not available.
